# Virtual Reality for Delirium Prevention in Intensive Care Unit Nurses and Mechanically Ventilated Patients: Descriptive Qualitative Study

**DOI:** 10.2196/91194

**Published:** 2026-10-23

**Authors:** Jiaxin Li, Liming Li, Mengjuan Jing, Huanmin Xing, Huijie Zhao

**Affiliations:** 1 College of Nursing and Health, Henan University, North Section of Jinming Avenue, Longting District, Kaifeng, Henan Province, 475004, China, 86+18803781880; 2 Nursing Department, Henan Provincial People's Hospital, Zhengzhou, Henan, China; 3 Department of Critical Care Medicine, Henan Provincial People's Hospital, Zhengzhou, China

**Keywords:** intensive care unit, mechanical ventilation, delirium, virtual reality, qualitative research

## Abstract

**Background:**

Patients in the intensive care unit (ICU) who are on mechanical ventilation are at high risk of developing delirium. Virtual reality (VR) technology, as an emerging intervention method, shows promising application prospects in delirium prevention, but its specific experience in clinical practice still requires in-depth exploration.

**Objective:**

This study aims to explore the real experiences of ICU nurses and mechanically ventilated patients in participating in VR delirium prevention, with the aim of providing practical evidence for the optimization and application of this technology in clinical practice.

**Methods:**

A descriptive qualitative research design was adopted. From March to June 2025, using purposive sampling, participants were selected from the internal medicine, surgery, central, and respiratory ICU departments of a tertiary hospital in Zhengzhou. A total of 73 patients on mechanical ventilation were screened. Among them, 49 were excluded due to not meeting the Richmond Agitation-Sedation Scale (RASS) or Barthel Index (BI) score criteria, 5 had delirium at the time of enrollment, 1 was excluded due to visual impairment, and 2 voluntarily withdrew during the study. Finally, 16 patients on mechanical ventilation and 16 nurses were included for semistructured interviews. Data analysis was conducted using the inductive content analysis method.

**Results:**

Through the analysis of the interview data, a total of 3 main themes and 14 subthemes were extracted. In terms of perceived benefits, patients experienced an immersive environment and a sense of empowerment driven by tasks, while nurses felt the convenience of resource integration, the intimacy of the nurse-patient relationship, and a sense of professional significance. In terms of perceived challenges, patients faced difficulties with the interaction interface and physical and mental obstacles, while nurses encountered difficulties in communication assessment and responsibility definition. In terms of perceived needs, patients expected personalized content design and continuity of nursing services, while nurses suggested differentiated content adjustment, comprehensive functional integration, and standardized safety management.

**Conclusions:**

The participants expressed a positive attitude towards the prevention of delirium based on VR and reported various perceived benefits, challenges, and needs. Future research should prioritize content personalization, functional integration, and safety management. Meanwhile, it is necessary to recognize the limitations of this technology in specific target populations. For those who do not meet the inclusion criteria, adaptive alternative intervention measures should be developed to promote the precise and sustainable application of VR-based delirium prevention.

## Introduction

Delirium is an acute brain syndrome characterized by impaired consciousness. It has an acute onset, a short course, and rapid symptom fluctuations [1]. In the intensive care unit (ICU), patients receiving mechanical ventilation are at a high risk of developing delirium, with an incidence rate of up to 50%‐80% [1,2]. It is also associated with prolonged mechanical ventilation, increased hospital stay, cognitive impairment, and an increased risk of adverse events (AEs) [3-5]. Prevention is of the utmost importance.

Its onset is related to various causes, such as neuroinflammation and an imbalance of neurotransmitters, as well as risk factors such as anxiety, pain, and sleep disorders [6]. The prevention strategies mainly include both pharmacological and nonpharmacological interventions. Common sedative and analgesic drugs have side effects and may even induce delirium [7-9], so nonpharmacological interventions are more favored. Measures such as sleep improvement [10], early activity [11], and music therapy [12] can intervene in the risk factors of delirium and improve the comfort and function of patients. However, traditional single measures have limited effects, and the implementation of bundled care faces the challenge of insufficient resources in the busy ICU environment [6,13]. Therefore, it is necessary to explore efficient and operational new prevention methods.

Virtual Reality (VR) technology integrates computer and sensing technologies, enabling the creation of immersive and interactive virtual environments, providing novel support and rehabilitation experiences for patients [14]. Studies have shown that VR shows potential in alleviating anxiety in conventional ICU patients [15], improving sleep [16], promoting activities [17], and enhancing cognitive training [18], and can reduce the incidence of ICU delirium and improve mechanical ventilation [19,20]. Moreover, evidence supports that VR can be safely used in patients with mechanical ventilation [21], and can effectively alleviate their anxiety [22] and improve cognitive function [23], and promote early activity [24].

At present, this field is still in the exploration stage, and there is a lack of in-depth understanding of the real experience of ICU nurses and mechanically ventilated patients using VR to prevent delirium. As implementers, the experiences of nurses affect the intervention effect; as users, the tolerance and acceptance of patients are the key to the application of the technology [25,26]. Qualitative research methods can deeply explore the feelings, needs and obstacles of participants, and providing rich details for understanding complex experiences [27].

Therefore, this study adopted a qualitative research design, using semistructured interviews to deeply explore the experiences and feelings of ICU nurses and mechanically ventilated patients regarding participation in the VR delirium prevention intervention. The aim was to provide practical evidence for optimizing the application of this technology in clinical settings.

## Methods

### Overview

This study aims to explore the experiences and feelings of ICU nurses and mechanically ventilated patients regarding the use of VR technology for the prevention of delirium, and to provide practical evidence for the application of VR technology in the prevention of delirium in the ICU.

### Design

This study used a descriptive qualitative research method, systematically collecting and analyzing textual data to deeply explore the characteristics of the phenomenon and its background. It is suitable for exploring emerging fields [28,29]. Data were collected through semistructured interviews, and their flexible nature helps to deeply uncover the true experiences of the respondents and obtain rich subjective experience data [30], thereby comprehensively presenting the cognition and practice of ICU nurses and patients regarding VR technology in preventing delirium. The research process followed the unified standard list for qualitative research reports, COREQ (Consolidated Criteria for Reporting Qualitative Research) [31], to ensure the quality and rigor of the study.

### Setting

From March to June 2025, this study was conducted in the central ICU, internal medicine ICU, surgical ICU, and respiratory ICU of a tertiary hospital in Zhengzhou City. The participants were selected using purposive sampling. After the patients were enrolled, they received the VR technology-based delirium prevention program until they were transferred out of the ICU or had their tubes removed. Each patient was matched with 1 nurse, and all the participants or their families signed the informed consent form. To ensure the homogeneity and continuity of the intervention and to avoid fragmentation and contamination effects, the VR intervention was implemented by the same nurse for each patient from the time of enrollment. During this period, this nurse was solely responsible for the VR intervention and did not participate in other bedside care to ensure the integrity of the nurse’s participation and the accuracy of the interviews. All nurses received unified VR technology and delirium management training. AE refers to any adverse medical events, unexpected injuries, or abnormal clinical signs experienced by the study participants. The determination of an AE does not depend on whether there is a causal relationship with the study device, whether it is foreseeable, whether medical intervention is required, or whether there are serious consequences [32]. For digital intervention schemes that combine software and hardware, the physical and mental adverse manifestations of the participants and the technical problems caused by the equipment and clinical operation procedures need to be distinguished; the former belongs to subjective physical and mental discomfort induced by digital intervention and falls within the category of AEs, while the latter are technical problems such as program freezing, network interruption, and abnormal hardware compatibility, which do not constitute AEs and are only recorded as information on the feasibility of the study. Only when equipment failure subsequently leads to physical and mental damage to the participants, can this secondary damage be determined as an AE [33].

The VR intervention uses the domestic PICO 4 device (PICO, ByteDance). It is divided into 4 modules each day at 9 AM: immersive relaxation (5 minutes), cognitive training (15 minutes), and breathing training (8 minutes); at 8:30 PM, there is mindfulness meditation (5 minutes) and sleep experience optimization (20 minutes). Immersive relaxation leads them to explore scenes such as colorful oceans, spring cherry blossoms, and grassland pastoral; interactive functional training trains patients’ cognitive functions through fun mini-games such as the fun farm and the detective of basketball; forest breathing, starlight aurora breathing, and mystery of the universe breathing assist patients in conducting breathing training; mindfulness meditation allows patients to follow the guidance to meditate in natural soundscapes such as the garden cabin, courtyard drizzle, and dawn of frosty mist; sleep optimization experience creates scenes such as a French fireplace snow cabin, urban rainy night café, and snow country train, combined with white noise, to help patients optimize the sleep experience (the specific process of the plan is shown in Figure 1).

**Figure 1.**
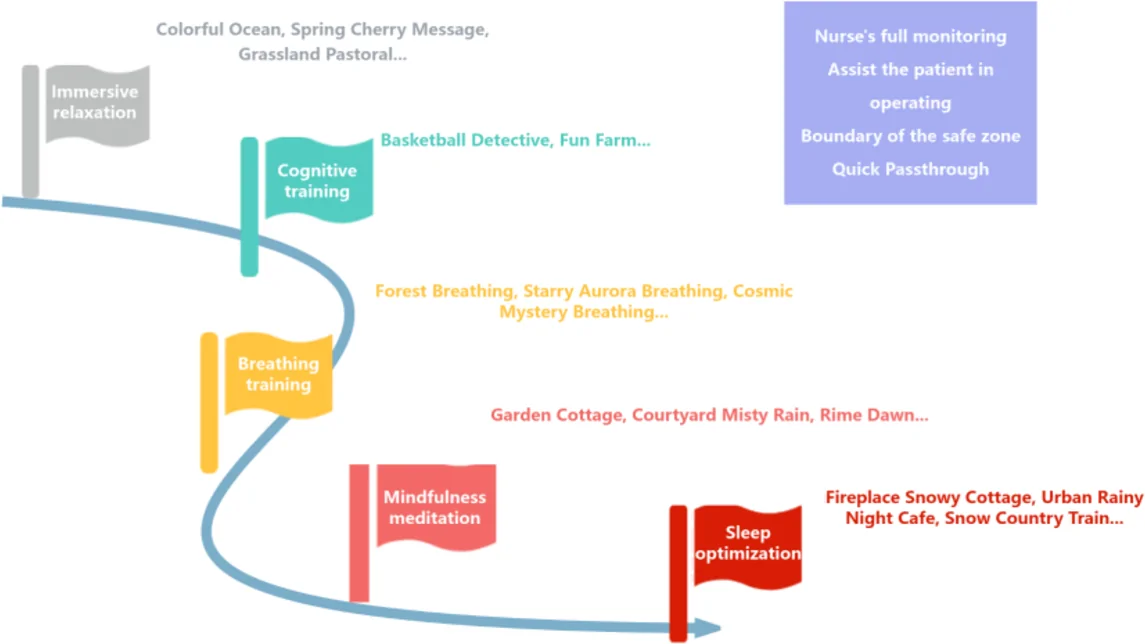
Virtual reality delirium prevention plan diagram.

In terms of safety measures, nurses provide continuous supervision and assistance to patients during the operation; the Pico equipment is equipped with a safety zone boundary indication (a blue barrier will appear when the body or handle touches the safety boundary, and the view will switch to the real world if it exceeds the safety boundary) and a quick passthrough function (the nurse or patient can double-click on the side of the helmet to switch the helmet’s view to the real world), to ensure patient safety.

### Participant Recruitment

This study selected the participants using purposive sampling in the ICU of the aforementioned hospital from March to June 2025.

Inclusion criteria for ICU nurses were as follows: (1) having received training in delirium care before participating in this study and are capable of independently conducting delirium prevention or care for mechanically ventilated patients; (2) having received training related to VR technology and can proficiently use VR equipment; (3) having been included using the maximum difference sampling method from different years of work experience, but with ICU work experience of ≥3 years and different job titles, so that the nurses participating in the study have different levels of delirium management experience; and (4) having voluntarily participated and signed the informed consent form. Exclusion criteria were not having been on duty during the study period and not being current nurses of this hospital.

Inclusion criteria for patients undergoing mechanical ventilation were as follows: (1) ICU patients; (2) first-time patients who underwent invasive mechanical ventilation, and the expected duration of invasive mechanical ventilation was≥48 hours; (3) age of ≥18 years; (4) a Richmond Agitation-Sedation Scale (RASS) score of 0 to +1 (patients within this range are awake or in a mild state of restlessness and can understand and follow instructions) and Barthel Index (BI) of ≥40 points (this score range ensures that the patients have the physical strength and activity ability to complete VR interactive actions); (5) no delirium at the time of enrollment; and (6) voluntary participation and signing of informed consent. Exclusion criteria were as follows: (1) patients with cognitive impairment or mental disorders who cannot cooperate; (2) severe visual or hearing impairment; and (3) various activity contraindications, such as severe cardiac or pulmonary insufficiency, recent major surgery or trauma, intracranial lesion, uncontrolled endocrine or metabolic disorders, etc.

This study used the maximum difference sampling strategy within the framework of purposive sampling. It purposefully included nurses of various ages, years of experience, professional titles, and ICU types, as well as patients of different ages, educational backgrounds, ICU types, and diseases (Table 1). Considering that participants from different backgrounds have diverse experiences and perceptions regarding VR delirium prevention, their acceptance of VR technology, immersive experience, and intervention needs may also vary. Therefore, this sampling method aims to obtain diverse perspectives in order to enhance the transferability of the research results. The determination of the sample size is based on the criterion of data saturation. That is, when the same topic is repeatedly encountered in 3 consecutive interview materials, and the newly collected data no longer provide additional information or details to enrich the existing topics, it is considered that the data have reached saturation.

**Table 1.** General information of the study participants (N=32).

Characteristics	Nurse (n=16), n (%)	Patient (n=16), n (%)
Age (years)		
18-29	4 (25)	2 (12.5）
30-39	6 (37.5）	2 (12.5）
40-49	4 (25)	4 (25）
50-59	2 (12.5)	5 (31.25）
≥60	—^a^	3 (18.75)
Gender		
Men	5 (31.25）	9 (56.25)
Women	11 (68.75)	7 (43.75)
Marital status		
Unmarried	3 (18.75)	1 (6.25)
Married	12 (75)	11 (68.75)
Divorce	1 (6.25)	3 (18.75)
Widowed	—	1 (6.25)
Educational background		
Primary school and below	—	7 (43.75)
Junior high school	—	5 (31.25)
High school	—	2 (12.5)
Bachelor’s degree	10 (62.5)	2 (12.5)
Master’s degree or above	6 (37.5)	—
Type of ICU^b^		
Medical ICU	3 (18.75)	3 (18.75)
Surgical ICU	4 (25)	5 (31.25)
Respiratory ICU	4 (25)	4 (25)
Central ICU	5 (31.25)	4 (25)
Professional title		
Junior professional title	5 (31.25)	—
Intermediate professional title	7 (43.75)	—
Senior professional title	4 (25)	—
Experience of working in the ICU (years)		
3-10	5 (31.25)	—
11-20	7 (43.75)	—
21-30	3 (18.75)	—
31-40	1 (6.25)	—
Employment status of patients		
Full-time job	—	10 (62.5)
Part-time job	—	2 (12.5)
Retired	—	3 (18.75)
Unemployed	—	1 (6.25)
Patient’s disease type		
Respiratory system diseases	—	3 (18.75)
Circulatory system diseases	—	4 (25)
Digestive system diseases	—	4 (25)
Neurological diseases	—	2 (12.5)
Urinary system diseases	—	3 (18.75)
Length of stay in the ICU (days)		
＜7	—	2 (12.5)
7-14	—	7 (43.75)
＞14	—	7 (43.75)

a Not applicable.

b ICU: intensive care unit.

### Data Collection

This study established a 7-member research team consisting of 1 associate professor (HZ) from the nursing college, 2 ICU nurse managers (MJ and HX), 2 ICU nurses, and 2 nursing graduate students (JL and Yuhao Zhao). All of them possess knowledge of delirium management and have received training in qualitative research.

The interview outline was initially drafted by 2 ICU nurse managers (MJ and HX) through a literature review, group discussion, and expert guidance. It was then optimized and finalized after preliminary interviews with 2 nurses and 2 patients. The interviews were conducted by 2 nursing graduate students (JL and Yuhao Zhao) with experience in delirium-related research and familiarity with delirium management. The interview outline is shown in Table 2.

**Table 2.** Outline of interviews with mechanically ventilated patients in ICU^a^ and nurses.

Interviewee	Specific issues
Patient	What is your understanding of VR^b^ technology? How did you feel when experiencing the VR device? What psychological and physiological changes did you experience before and after using the VR device, and what impacts did it have on you? What problems or barriers did you encounter during the experience? From a patient’s perspective, what suggestions or improvement ideas do you have for the application of VR technology?
Nurse	What is your understanding of VR technology? How do you perceive the application of VR technology in preventing delirium among mechanically ventilated patients in ICU? What are the main advantages of VR technology? What problems or barriers do you think exist in the application of VR technology for delirium prevention in mechanically ventilated patients in ICU? From a health care perspective, what suggestions or improvement ideas do you have for the application of VR technology in delirium prevention among mechanically ventilated patients in ICU?

a ICU: intensive care unit.

b VR: virtual reality.

After the patients complete the VR intervention (either extubation or transfer out of the ICU), a semistructured interview is conducted with the patients and the matching nurses within 30 to 60 minutes. To reduce memory bias, the interview arrangement is as follows: for patients who have been extubated in the ICU, it is conducted beside the curtain at the bedside (with a “Interview in progress, please do not disturb” sign hanging outside the curtain, strictly restricting the entry of irrelevant personnel to minimize the risk of external interference and information leakage); for those who have been transferred to the general ward and have been extubated, it is conducted in the meeting room of the ward; for patients who have been discharged from the ICU but have not been extubated, communication is carried out through writing or typing; the interviews with the nurses are all conducted in the department meeting room.

Two graduate students (JL and Yuhao Zhao) majoring in ICU nursing served as interviewers. Before the interview, they communicated with the participants, agreed on the interview time and location, and informed them of the interview process. During the interview, the researchers asked questions based on the outline and made flexible follow-up inquiries, recorded both verbal and nonverbal information, and took notes and made audio recordings simultaneously. If the interviewee became emotionally agitated, they would pause and adjust. The audio data was encrypted and stored, accessible only to the research team. On the day of the interview, 2 ICU nurses (HX and Hao Li) transcribed the text, and after the participants confirmed it, they summarized and analyzed it. When there were no new themes in 3 consecutive sets of data, it was determined that data saturation had been reached. To reduce the potential bias that might arise from both the interviewer and the interviewee having the same background in ICU nursing, the following control measures were adopted in this study: (1) both of the 2 nursing graduate students (JL and Yuhao Zhao) who conducted the interviews received systematic training in qualitative research and conducted preinterview exercises before the formal interviews, which strengthened their neutral stance and reflective awareness and prevented them from bringing their own clinical experience or preconceived opinions into the interview process; (2) the interviews strictly followed a semistructured outline, and the interviewers were required to avoid leading or judgmental language and encourage the interviewees to freely and fully express their true feelings; and (3) after each interview, each interviewee would check the interview records to ensure that the collected information accurately reflected their true intentions rather than the subjective interpretation of the interviewers.

### Data Analysis

This study used the inductive content analysis method. Two nurses independently (HX and Hao Li) coded the data, and a nonclinical background associate professor reviewed it to prevent the influence of the analysts’ backgrounds on the interpretation of the results. The team conducted 3 rounds of discussions to resolve differences and reach consensus, ensuring the accuracy and consistency of the analysis results. This method does not require a predefined theoretical framework and can systematically summarize concepts and themes from the original data, which is suitable for exploring complex phenomena and helps to deeply understand the real experiences of the research object [28-30]. The specific analysis steps are as follows:

1. Data Preparation: Transcribe the interview recordings into text, perform deidentification to protect privacy, and read through the entire text to grasp the overall content.
2. Line-by-line Coding: Analyze the text line by line, identify and mark meaningful content units, and form initial codes. For example, the patient’s description, “feeling as if I were on the beach, without the cold machines,” is coded as “immersive relaxation.”
3. Cluster Subthemes: Group and integrate similar initial codes to form more general sub-themes. For example, the codes “immersive relaxation” and “multisensory soothing” can be classified as “immersive VR situational experience.”
4. Extract Core Themes: Further integrate the subthemes, abstract, and refine the core themes that can reflect the essence of the phenomenon. For instance, through the analysis of multiple subthemes, the core theme “benefits of perceived involvement in VR delirium prevention” can be ultimately extracted.
5. Verification and Improvement: Cross-verify the text content with nonverbal information recorded in the interviews (such as expressions and tone) to enhance the credibility of the results.

The research team handled coding disputes through a structured 3-round discussion: in the first round, each presented the basis for the coding; in the second round, they focused on the main points of disagreement and delved deeper into the original text for discussion; in the third round, they aimed to reach consensus, based on the majority opinion and the research purpose, to determine the final coding and themes.

After inductively extracting themes and subthemes from interview data, the research team used the Systems Engineering Initiative for Patient Safety (SEIPS) model as a theoretical framework to organize and interpret findings [34]. The SEIPS model describes a work system that consists of 6 interacting elements, named Persons, Tasks, Tools and Technology, Organization, Internal Environment, and External Environment, and holds that work process outcomes are shaped by interactions and adaptations among these elements. In this study, extracted themes and subthemes were mapped onto the 6 SEIPS elements to systematically identify facilitating factors and implementation obstacles for VR-based delirium prevention in the ICU.

### Rigour

This study adheres to the rigorous framework for qualitative research proposed by Guba and Lincoln [35]. To ensure the quality of the research, the following aspects were implemented: reliability was ensured through independent transcription and coding by 2 individuals (HX, Hao Li and Huijie Zhao) and review by a third party; credibility was achieved by extending the interview duration, reaching consensus with the research team, and conducting triangulation of data with field notes; transferability was enhanced through maximum difference sampling; confirmability was ensured by fully preserving the data, using software for analysis, and following a strict bottom-up process to avoid interference of theoretical presuppositions in data analysis.

### Ethical Considerations

This study was conducted in accordance with the Helsinki Declaration and was approved by the Bioethics Committee of Henan University on September 2, 2024 (approval number: HUSOM2024-434). All participants or their legal representatives were fully informed of the study purpose, process, potential risks and benefits, as well as the right to withdraw at any time without affecting their clinical treatment, and signed a written informed consent form before enrollment. All interview data were anonymized to protect the identities of the participants. Each participant was assigned a unique code (Patient P1-P16, Nurse N1-N16), and the transcripts and final reports do not contain any personal identification information. The audio files were stored on a device with password protection, accessible only to the research team. This study did not provide any economic or other forms of compensation to the participants.

## Results

### Overview

The study evaluated a total of 73 ICU patients aged 18 years or older who underwent invasive mechanical ventilation for the first time and whose expected duration of invasive mechanical ventilation was≥48 hours. Among them, 49 patients were excluded due to noncompliance with the RASS score and BI score criteria. 5 patients had delirium at the time of enrollment, 1 patient was excluded due to visual impairment, and 2 patients voluntarily withdrew during the study. In total, 32 participants were included in this study, including 16 ICU patients undergoing mechanical ventilation and 16 ICU nurses. During the VR intervention process, some patients experienced adverse physical and mental reactions, including dizziness, anxiety, and tension induced by the intervention, fatigue, and physical discomfort. These symptoms could be alleviated by suspending the VR intervention, and no additional clinical treatment was required. According to the definition [32,33], the above situations were classified as AEs. The study also recorded several process obstacles, mainly including clinical diagnosis conflicts and difficulties in equipment operation. These obstacles did not directly harm the patients, but led to the premature termination of the VR intervention. The combination of these 2 types of problems resulted in 23 VR interventions failing to be completed as planned. General information of the participants is shown in Table 1, and the records of VR intervention compliance and adverse experiences are shown in Tables 3 and 4. Based on the interview data, a total of 3 themes and 14 subthemes were extracted (Figure 2).

**Table 3.** VR^a^ intervention compliance and experience records.

Indicator	Result	Notes
Total hospitalization days of the patients (days)	192	—^b^
Number of daily plans (times)	2	—
Total number of plans (times)	384	—
Actual completion times (times)	361	The overall compliance rate was 94.0%.
Average duration of each use (minutes)	24	—
Number of incomplete times (times)	23	It was not completed due to both AEs^c^ and process obstacles.

a VR: virtual reality.

b Not applicable.

c AE: adverse event.

**Table 4.** VR^a^ negative experience records.

Types of negative experience	Number of events	Number of affected patients	Notes
AEs^b^			
Vertigo	4 times	1 patient	The symptoms will subside on their own after stopping VR use, and no medical intervention is required.
Nervousness and anxiety	3 times	3 patients	The symptoms will subside on their own after stopping VR use, and no medical intervention is required.
Fatigue or physical discomfort	7 times	5 patients	The symptoms will subside on their own after stopping VR use, and no medical intervention is required.
Process obstacles			
Clinical diagnosis and treatment conflicts	6 times	5 patients	Conflicts in clinical work such as suctioning and blood gas sampling
Difficulty in operating the equipment	3 times	3 patients	Problems with executing operations

a VR: virtual reality.

b AE: adverse event.

**Figure 2.**
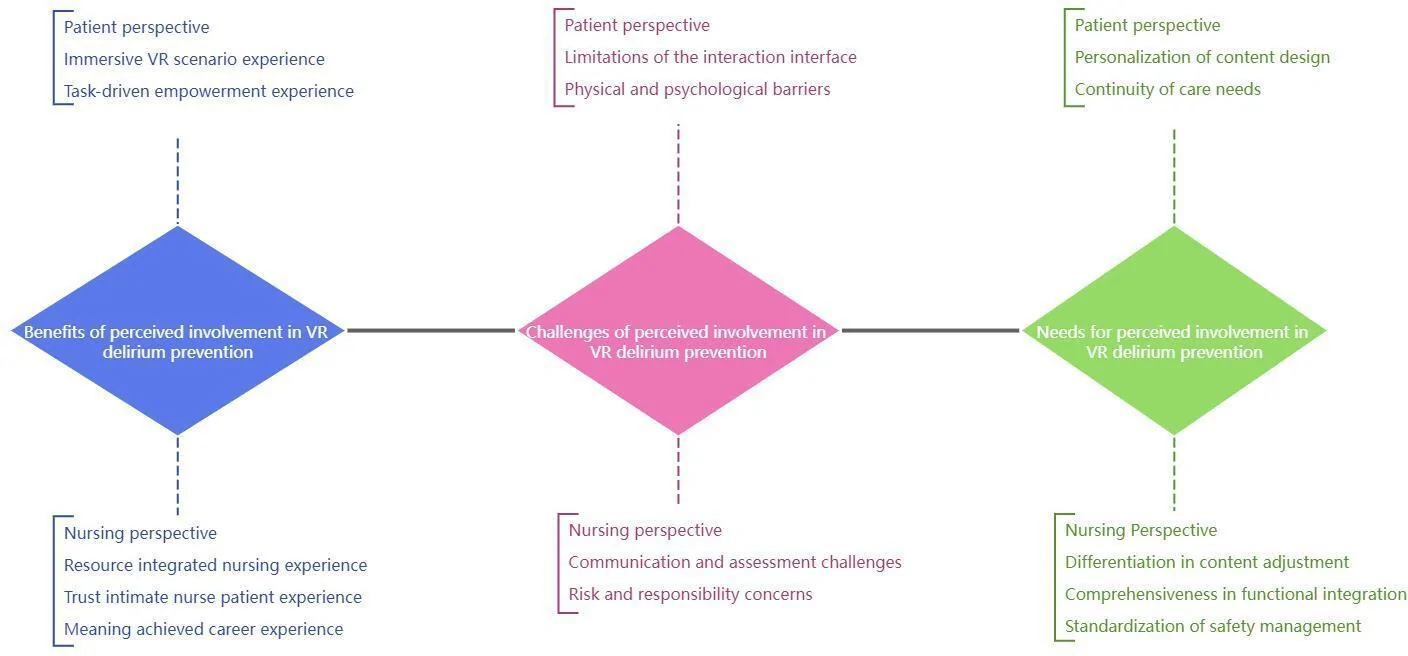
Themes and subthemes in the research. VR: virtual reality.

### Benefits of Perceived Involvement in VR Delirium Prevention

#### Patient Perspective

##### Immersive VR Scenario Experience

Most patients undergoing mechanical ventilation reported that the VR delirium prevention intervention provided them with multisensory relaxation and immersive experiences, breaking the monotony of the hospital ward. Through the interactive functions of VR, patients could actively participate in simulation activities and feel that their physical functions had been exercised.

Wearing the VR device, I can go to a peaceful beach. In front of me is the illuminated sea surface, and in my ears are the sounds of the waves and the chirping of birds. This temporarily makes me forget my illness and my mood becomes much more relaxed. Especially when I have a tube inserted and am often thirsty, the mini-game in the VR where you open the refrigerator and see the cold air and beverage images makes me feel extremely refreshing and quenched.

[P8]

I listened to the sounds of the fireplace and the rain in the VR, and followed the voice to empty myself in the forest. The environment became quiet, and I could fall asleep unconsciously. I became less sensitive to the alarm sounds and conversations around me. The small games played through the controller allowed me to move even when the tubes were plugged in, and my arms felt less stiff; the fruit-picking game made my mind more agile, and I could concentrate without daydreaming.

[P9]

##### Task-Driven Empowerment Experience

Most patients believe that the game settings and real-time feedback in the VR environment enable them to immerse themselves more deeply in the experience, allowing them to gain a sense of freedom and control that is different from reality.

In VR, it made me feel that I could still do many things, unlike before when I could only lie down. Every time I completed a small task, there would be a reward prompt on the screen. That sense of achievement made me very happy. Although I knew it was a simulation, seeing the training data improve little by little, I felt that I was getting closer to a normal life, no longer a passive patient receiving treatment.

[P11]

### Nursing Perspective

#### Resource-Integrated Nursing Experience

Most nurses believe that VR has overcome the limitations of time and space by integrating multiple measures for preventing delirium, and it is efficient and convenient to use. Compared with traditional bundled measures, nurses have felt that VR has alleviated their workload in delirium prevention.

In the past, when preventing delirium, we were under great mental stress. We had to prevent tube removal, chat with the patients, adjust the lighting, play music, and conduct cognitive training. When there were fewer staff, it was even more chaotic. Now with VR, patients can listen to the sound of waves and the virtual wind. Their vital signs are stable, and they cooperate with the treatment. One device replaces multiple operations, saving manpower and time. This enables us to focus more on observing the patient’s condition and optimizing the care, which is beneficial for both the nurses and the patients.

[N6]

#### Trust Intimate Nurse-Patient Experience

VR technology provides a new platform for nurse-patient interaction. Nurses participate throughout the patient’s experience, achieving close and continuous interaction, thus making the nurse-patient relationship feel more intimate.

Previously, nursing work was mostly routine operations, with limited communication with patients. Now, with the help of VR, we can assist patients in wearing the equipment, select appropriate scenarios based on their emotions and needs, and then provide companionship, guidance, and encouragement. This enables us to deeply understand their feelings, which has strengthened the trust between us and the patients and made our relationship more intimate.

[N1]

#### Meaning Achieved Career Experience

Most nurses stated that the application of VR technology has enabled them to transform from traditional disease caregivers to comprehensive protectors of patients’ physical and mental health, allowing them to have a deeper understanding of the value and significance of their work.

Previously, tasks such as administering injections, changing dressings, and monitoring clinical progress were important, but they were more like following medical instructions, lacking warmth. Wearing VR devices for patients was not simply handing something over. Seeing them experience it and nodding or giving a thumbs-up, I felt a great sense of achievement. At the same time, VR also exposed me to cutting-edge nursing concepts, and from it, I gained new professional knowledge and skills, allowing me to truly understand that our work is not only about saving lives, but also about helping patients regain hope. This is the true meaning of nursing.

[N13]

### Challenges of Perceived Involvement in VR Delirium Prevention

#### Patient Perspective

##### Limitations of the Interaction Interface

Some patients reported that the interactive interface of the VR system affected their overall experience of using it.

Looking at all the buttons and menus on this device, I’m totally lost. I don’t know how to operate it. I memorize the steps, but the moment I turn around, I forget everything.

[P6]

When the scenes change too rapidly, one may feel dizzy. Some games are too realistic. For instance, in the basketball shooting game, I felt as if that basketball was really going to hit me, which was a bit scary. Some scenes are in the air, and I have a bit of acrophobia.

[P5]

##### Physical and Psychological Barriers

Patients undergoing mechanical ventilation are bedridden for a long time, resulting in physical weakness, reduced limb strength, and mobility. When using VR equipment, they are prone to fatigue and consume a lot of energy. Some patients are unfamiliar with VR technology and are worried that the equipment might cause harm to their bodies; thus they have reservations and are very nervous when using it.

I’m lying in a hospital bed and haven’t exercised in a long time. I tried some scenes in the VR, but found that I had to constantly raise my hands and turn my head. However, my body was too weak and I couldn’t manage to follow the movements.

[P4]

When performing the movements and adjusting the helmet, I was extremely cautious, fearing that I might pull the tubes. During my first attempt, all I could think about was ’Will the tubes in my nose be damaged, affecting the machine’s air supply?' When using it, my body unconsciously tensed up.

[P13]

### Nursing Perspective

#### Communication and Assessment Challenges

The nurses reported that patients with intubation had difficulty accurately conveying the complex emotions experienced in the VR environment, fearing that misunderstandings would affect the implementation of the intervention; moreover, most of the nurses stated that it was difficult to quantitatively assess the patient’s experience and changes in the risk of delirium, which would hinder the precise application of VR in the prevention of delirium.

Every time I try to understand the patient’s feelings, I can only rely on observing their expressions and subtle gestures. Sometimes the patient shakes their head, and it’s hard to tell whether they are looking at different scenes or expressing dissatisfaction. I’m afraid of misunderstanding, and not only can’t help them, but it even makes them feel worse.

[N7]

The problem is that currently there are no specific tools or guidelines available to assess the effectiveness of VR use in ICU patients. We have to rely on our own observations and guesswork to determine how patients are experiencing it and whether the risk of delirium caused by VR has decreased.

[N4]

#### Risk and Responsibility Concerns

Some nurses pointed out that although VR technology is already mature, when it is used as a medical device in the ICU, there are concerns about legal risks due to the lack of clinical guidelines and clear responsibility definitions.

We are quite optimistic about VR technology. However, when it is used in the ICU, the rules and responsibilities are not clear. We don’t know who should be held responsible if something goes wrong. Both the nurses and the hospital could be involved. This uncertainty makes us feel very unsure.

[N13]

### Needs for Perceived Involvement in VR Delirium Prevention

#### Patient Perspective

##### Personalization of Content Design

Most patients expect VR content to be more diverse and personalized, in order to alleviate the monotony of the hospital ward. They have a greater demand for emotional interaction with family members, and hope that VR can provide immersive family scenarios or recreate cherished memories, thereby alleviating the emotional void caused by isolation.

If there were furry cartoon animals or beautiful Chinese-style scenes in VR, just looking at them would make one’s mood improve.

[P17]

I really want to see the familiar rural courtyard and the villagers working in the fields. I even more desire to return home and see the family members having meals and chatting together. Even just hearing their voices would be extremely comforting.

[P5]

##### Continuity of Care Needs

Most patients hope to extend VR technology into continuous care, in order to obtain a continuous relaxation experience and psychological support.

While I was in the ICU with the tubes, VR helped me divert my attention and do activity training. After being discharged from the ICU, the tubes were still there, and I was always worried. I hope the VR program in the ICU can be continued in the general ward, at least until the tubes are removed, which can alleviate anxiety and also contribute to recovery.

[P2]

### Nursing Perspective

#### Differentiation in Content Adjustment

Most nurses suggest that VR content should be designed differently based on the age and cultural background of the patients, in order to better match their actual situations.

It is suggested that VR content should be categorized based on age groups and cultural backgrounds. Young men can have more elements of science fiction and espionage; female patients can focus on intangible cultural heritage and fashionable art. Elderly people can concentrate on opera, history, and tea drinking and playing chess in tea houses. The content should be developed in line with the regional cultural background of the patients, such as the scenery of the Jiangnan water towns or the folk customs of the northwest. At the same time, the duration of the content should be controlled to avoid patient fatigue.

[N13]

#### Comprehensiveness in Functional Integration

Some nurses stated that, based on the characteristics of patients with tracheal intubation, nonpharmacological preventive measures should be systematically integrated into VR. VR not only needs to provide a relaxing experience, but also should incorporate educational and motivational elements in order to better support the long-term prevention and rehabilitation of patients.

The application of VR in the ICU can be more comprehensive. In terms of training, more virtual scenarios can be designed so that patients can perform breathing exercises in different scenarios. This offers more options and is easier for them to stick to. Additionally, vivid and intuitive animations can be used to perform the principles of the disease and the treatment process for them, which will help them understand better. The effect will be better than watching ordinary videos.

[N8]

#### Standardization of Safety Management

The ICU environment has high requirements for equipment safety. Nurses believe that the introduction of VR technology poses risks related to equipment and fire safety; therefore, strict management measures should be implemented.

The ICU environment is quite special, with high oxygen concentration and a large number of multi-drug resistant patients. If VR equipment is introduced, strict control over disinfection procedures and consumable management is necessary; otherwise, cross-infection could easily occur. Fire safety also needs to be considered. If the equipment gets hot or is charging, and if a circuit short-circuit were to happen, it would be a big problem. These aspects of wearing, monitoring, and disinfection could all increase our workload.

[N14]

The correspondence between the extracted themes and the elements of the SEIPS model [34] is summarized in Table 5.

**Table 5.** Correspondence of elements in the SEIPS^a^ model.

SEIPS model components	Promoting factors	Obstacle factors
Persons	Patient: immersive situational experience brings emotional relaxation and psychological empowerment (3.1.1) Nurse: resource integration sense, nurse-patient intimacy, and professional achievement sense (3.1.2)	Patient: limited physical capacity, dizziness, anxiety, difficulty in operation, and concerns about the tubes (3.2.1) Nurse: difficulties in communication, lack of dedicated assessment tools, and concerns about liability risks (3.2.2)
Tasks	VR^b^ integrates multiple nonpharmacological interventions such as relaxation, cognitive training, breathing exercises, meditation, and sleep optimization (2.2)	Conflicts with clinical operations such as suctioning and blood gas sampling (Tables 3 and 4); wearing, monitoring, and disinfecting the equipment adds additional workload (3.3.2)
Tools and technology	PICO 4 safety zone boundary indicators, quick perspective function, and multisensory virtual environment (2.2, 3.1.1)	Interface is complex and some scenarios cause discomfort, content lacks personalization, and function integration is insufficient (3.2.1, 3.3)
Organization	Unified specialized training and full-time nurses ensure the continuity of intervention throughout the process (2.2)	Lack of standardized operation procedures, unclear definition of responsibilities, and absence of continuous care support systems (3.2.2, 3.3.1)
Internal environment	The VR virtual environment can go beyond the physical space limitations of the ICU^c^ ward and create an immersive relaxation environment (3.1.1).	The high-oxygen environment in the ICU has strict requirements for the electrical safety of equipment; the presence of multidrug-resistant bacteria imposes strict demands on disinfection procedures; and tubing restricts physical movement (3.2.1, 3.3.2).
External environment	The policy environment is relatively lenient, allowing for exploratory clinical applications.	There is a lack of industry guidelines and regulatory norms for ICU VR applications in China; and the equipment access and charging procedures are unclear (3.2.2).

a SEIPS: Systems Engineering Initiative for Patient Safety.

b VR: virtual reality.

c ICU: intensive care unit.

## Discussion

### Principal Findings

Based on the SEIPS model framework [34] elaborated in the methodology section, this study analyzed the perceived promoting factors and obstructive factors for the promotion of VR delirium prevention intervention among ICU nurses and mechanically ventilated patients, focusing on 6 interacting elements: personnel, tasks, tools and technologies, organization, internal environment, and external environment. The model indicates that the effectiveness of the application of new technologies is not only determined by the technical attributes of the tools themselves, but also depends on whether the tools can effectively adapt to the operator’s capabilities, task processes, the environment they are in, and the supportive organizational system. The higher the degree of adaptation, the better the final implementation effect.

### Perceived Benefits and Limitations in Human-Technology Interaction

In the SEIPS model, human and technological tools are 2 closely integrated elements, and the quality of their interaction directly affects the technology application experience [34]. In this study, the positive interaction between humans and technology resulted in perceived benefits. VR, through multisensory virtual scenes, shifted patients’ attention away from pain and monitoring noises, creating a perceptual space isolated from the physical environment of the ICU, making patients feel emotionally relieved and at ease. The immediate feedback from gamification interaction enabled patients to gain a sense of active control while lying passively, compensating for the loss of autonomy due to illness, which is consistent with previous findings that VR can alleviate anxiety in ICU patients [22]. What the nurses experienced was not the sequential execution of traditional bundled care, but a more continuous process; the full-time companionship also generated scarce interaction opportunities in routine care, allowing nurses to perceive an intimate nurse-patient relationship and a sense of achievement; this is consistent with the conclusion proposed by Dailah et al [36] that digital tools can enhance the satisfaction of the nursing profession. These indicate that when the technical functions are well adapted to human needs, the interaction between human and technological elements can produce positive effects.

However, there are also limitations in the interaction between the two, and the obstacles stem from the mismatch between human physiological, psychological, and cognitive characteristics and technological design. Physically, patients’ long-term bed rest leads to muscle strength decline and limited mobility, while VR interaction requires head rotation and upper limb movement, and the mismatch between ability and demand results in fatigue and limited participation; some patients’ dizziness may be due to the physical settings of the device screen not matching the patient’s line of sight focus, but this reaction is tolerable for most patients and does not cause safety issues [37]; psychologically, patients’ concerns about pipeline safety, the way of wearing the equipment close to the tracheal tube, trigger physical and mental tension. Additionally, interaction interfaces exceeding their cognitive levels lead to operational difficulties, and the lack of personalization and insufficient function integration reflect the adaptability limitations of VR technology for patients from different cultural backgrounds. Among the 73 patients in this study, 49 (67.12%) cases were excluded due to not meeting the standards for consciousness or activity ability. This high exclusion rate clearly defines the applicable boundaries of VR intervention; that is, VR is more suitable for mechanically ventilated patients with clear consciousness and certain activity ability, and is more difficult to implement for patients with deep sedation or severely impaired functions, suggesting that the disease stage plays a regulatory role in the interaction between humans and technology.

### Implementation Process Guarantees and Barriers in Task and Organization

In the SEIPS model, tasks and organizational elements are mutually dependent [34]. The sequence, complexity, and resource requirements of tasks are constrained by organizational scheduling, personnel allocation, and institutional regulations, while organizational support affects the quality of task completion. In this study, both provide implementation support. At the task level, VR integrates multiple nonpharmaceutical interventions into the same device, facilitating nurses’ unified execution; at the organizational level, unified specialized training and full-time nurses’ continuous responsibility ensured the continuity of task execution. Both are important conditions for achieving a 94.0% compliance rate in this study, demonstrating their role in the implementation effect.

However, in clinical application, there are also contradictions between task characteristics and organizational resources. The task time is fixed as either morning or afternoon, which conflicts with the unpredictable nature of the organizational environment. The fixed-time characteristics of VR tasks and clinical operations such as suctioning and blood gas collection compete with each other. Among the 23 times the intervention was not completed in this study, 6 times it was interrupted due to this reason. This is similar to the mechanism that makes bundle care difficult to implement in busy ICUs. However, VR intervention has advantages such as equipment integration and strong operability, making it more flexible in terms of time usage [6,13]. There is a contradiction between the additional workload of the task and the organization’s human resource allocation. Equipment preparation, wearing monitoring devices, and disinfection after use increase the complexity of the task. When incorporated into the routine process, it will compete for limited human resources. The organizational support lags behind the task requirements. Specifically, there is a lack of effective communication tools for patients with mechanical ventilation, making it difficult for patients to obtain timely information; there is a lack of standardized operating procedures, resulting in no norms for task execution; the responsibility attribution is unclear, causing nurses to have legal-level risk concerns; and there is a lack of supporting continuity care systems. This is essentially an inability of organizational elements to make adaptive adjustments to new task types. Consistent with the qualitative research by Thissen et al [38] on the implementation obstacles of the ICU computerized intensive care information system, the mismatch between task processes and organizational systems is the main resistance to the implementation of new technologies. In the future, the operation process of VR intervention in ICUs can be adjusted, personnel arrangements can be optimized to make it more in line with clinical needs, and the existing mismatch problems in the implementation process of new technologies can be improved.

### Physical Space and Policy Norms in Environmental Constraints

The internal environment in the SEIPS model, which refers to the physical characteristics of the workplace [34], has a facilitating effect on the implementation of VR, but there are also limitations that need to be considered. The facilitating effect is manifested in that the VR scene builds a virtual natural environment for patients through a combination of audio and visual means, compensating for patients’ need for environmental diversity, which is consistent with the findings in previous studies that VR improves the environmental experience of ICU patients [39]. However, due to the influence of the special clinical environment of the ICU, the application of VR requires strict management. Analyzing the reasons, the high-oxygen environment in the ICU ward combined with the slight heat generation during the operation of the VR headset poses potential fire safety hazards, which is also a concern for nurses in practical applications. Secondly, the ICU ward has the characteristic of multiple colonization of drug-resistant bacteria, and the VR equipment has the application requirement of multiple people sharing. This makes surface disinfection of the equipment an important part of infection control, and currently, there is no standardized operating specification for VR equipment in clinical practice, which to some extent increases the difficulty of clinical infection control. The study points out that the head-mounted display may become a carrier of microbial transmission in the medical environment, and the current classification and management requirements are still insufficient, suggesting the need to establish cleaning, disinfection, and reuse norms for VR equipment [40]. In addition, various treatment tubes such as tracheal intubation and monitoring leads left by ICU patients will limit the range of head and upper limb movements of patients. Therefore, VR scenes that require significant body coordination should consider the patient’s physical condition.

The external environment refers to the macro policies and regulatory factors outside the organization [34]. From the perspective of the external environment, there are currently no professional guidelines or regulatory norms for the application of VR technology in ICU scenarios. Garrett et al [41] believe that the lack of complete policies will affect the configuration willingness of medical institutions and is not conducive to the regular and sustainable implementation of VR technology in the ICU. Therefore, in the future, lightweight, easy-to-disinfect, and compatible with medical tubes dedicated VR hardware can be developed, and equipment cleaning and disinfection norms can be established, and industry standards can be improved to ensure the feasibility and standardization of VR intervention in the complex physical environment of the ICU.

### Limitations

This study is a single-center design, and there are certain limitations in the representativeness of the sample, which may affect the transferability of the results; future multicenter longitudinal studies can be conducted to more comprehensively evaluate the long-term effects of VR in different social and cultural contexts. At the same time, the inclusion criteria limit the participants to those who are conscious and have certain activity abilities among mechanical ventilation patients. Therefore, when extending the conclusion to patients with deeper sedation or poorer functional status, caution is needed; future simplified VR programs can be developed for the excluded subgroups. The adverse experiences reported in this study were all brief and mild subjective feelings, and no serious AEs occurred. However, relevant data still need to be collected in the future to establish a more complete safety assessment framework.

### Conclusions

This study, based on the experiences of patients and nurses, identified the perceived benefits, challenges, and demands of both in the prevention of VR delirium. Patients gained immersive experiences and a sense of task-driven empowerment; nurses shared the convenience of integrating nursing resources, experiencing a closer nurse-patient relationship, and professional significance. Both parties pointed out challenges such as insufficient technical adaptation, difficulties in communication and assessment, and ambiguous responsibility definitions. They also expressed diverse demands for personalized content, functional integration, and safety management. The exclusion rate of up to 67.1% indicates that VR intervention is only applicable to specific groups of people, not all ICU mechanically ventilated patients. These findings enrich the understanding of the application of VR technology in ICU delirium prevention from a qualitative perspective, suggesting that when promoting this technology in the future, attention should be paid to clinical individual differences and organizational support, establishing a screening process for matching patients and interventions, and formulating alternative intervention plans for those who are not suitable.

## Abbreviations

AE: adverse event
BI: Barthel Index
COREQ: Consolidated Criteria for Reporting Qualitative Research
ICU: intensive care unit
RASS: Richmond Agitation-Sedation Scale
SEIPS: Systems Engineering Initiative for Patient Safet
VR: virtual reality
